# Comparative analysis reveals similarities between cultured submandibular salivary gland cells and liver progenitor cells

**DOI:** 10.1186/2193-1801-3-183

**Published:** 2014-04-09

**Authors:** Olga S Petrakova, Vasiliy V Terskikh, Elena S Chernioglo, Vasiliy V Ashapkin, Evgeny Y Bragin, Victoria Y Shtratnikova, Inessa G Gvazava, Yuriy V Sukhanov, Andrey V Vasiliev

**Affiliations:** Faculty of Biology, Lomonosov Moscow State University, Leninskie Gory 1, bld. 12, Moscow, 119991 Russia; Koltsov Institute of Developmental Biology, Russian Academy of Sciences, Vavilov str. 26, Moscow, 119334 Russia; Belozersky Institute Moscow State University, Leninskie Gory 1/40, Moscow, 119991 Russia; Center of Innovation and Technology of Biologically Active Compounds and Their Applications, Russian Academy of Sciences, Gubkin str. 3/2, Moscow, 117312 Russia; Pirogov Russian National Medical Research University, Ostrovitianov str. 1, Moscow, 117997 Russia

**Keywords:** Gene expression, Liver cells, Submandibular salivary gland cells, Transcriptome

## Abstract

Mouse submandibular salivary gland cells and liver progenitor cells from long-term *in vitro* cultures with a high proliferation potential were side-by-side compared by methods of immunocytochemistry, quantitative real-time PCR, flow cytometry, and transcriptome analysis. The two cell types were found to be similar in expressing cell markers such as EpCAM, CD29, c-Kit, Sca-1, and c-Met. In addition, both cell types expressed cytokeratins 8, 18, and 19, alpha-fetoprotein, and (weakly) albumin. Unlike the liver cells, however, the salivary gland cells in culture showed high-level expression of cytokeratin 14 and CD49f, which was indicative of their origin from salivary gland ducts. Quantitative real-time PCR and deep-sequencing transcriptome analysis revealed similarities in the expression pattern of transcription factors between the two cell types. In this respect, however, the cultured salivary gland cells proved to be closer to exocrine cells of the pancreas than to the liver progenitor cells. Thus, ductal cells of postnatal submandibular salivary glands in culture show phenotypic convergence with progenitor cells of endodermal origin, suggesting that these glands may serve as a potential cell source for cellular therapy of hepatic and pancreatic disorders. The results of this study provide a deeper insight into the molecular features of salivary gland cells and may help optimize procedures for stimulating their differentiation in a specified direction.

**Electronic supplementary material:**

The online version of this article (doi:10.1186/2193-1801-3-183) contains supplementary material, which is available to authorized users.

## Introduction

Cell-based approaches to treatment of liver pathologies are at the forefront of research in medical biotechnology. However, despite certain success in laboratory studies, no safe and sufficiently effective techniques for practical application has been developed to date. Progress in this field is hampered by shortage in the tissue sources of the cells capable of transdifferentiation into endodermal cell types. Primary human hepatocytes or liver progenitor cells appear to be most suitable in this respect, but their availability is limited. Promising approaches to the cellular therapy of hepatic and pancreatic pathologies involve the use of induced multipotent stem cells differentiating into the endodermal lineage (Rambhatla *et al.*^2003^; Hay *et al.*^2008^; Mizumoto *et al.*^2008^; Cayo *et al.*^2012^; Cheng *et al.*^2012^) or patient-specific cells capable of such transdifferentiation (Bisgaard and Thorgeirsson 1991; Kordes *et al.*^2012^; Yi *et al.*^2012^).

Submandibular salivary glands can be a convenient source of autologous cells for cellular therapy. The biopsy of salivary gland cells (SGC) is a simple medical procedure, and the cells are easy to culture. Since postnatal submandibular salivary glands express some endodermal markers, such as alpha-fetoprotein and preproinsulin (Tsuji and Nagai 1993; Egea *et al.*^2000^), their ability to transdifferentiate into various endodermal cell types has been actively studied during the past decades (Okumura *et al.*^2003^; Sato *et al.*^2007^; Baek *et al.*^2012^). Although the endodermal origin of major salivary glands has not been confirmed (Rothova *et al.*^2012^), the submandibular gland cells possess a high potential for transdifferentiation in hepatic and pancreatic directions.

When cultured *in vitro,* SGC actively proliferate and express cytokeratin 19 and alpha-fetoprotein (Gvazava *et al.*^2011^). Cells expressing high levels of integrin α6β1 and c-Kit (surface markers of stem/progenitor SGC) isolated from normal rat submandibular salivary gland were found to maintain the morphology, proliferation activity, and multipotency of stem cells for up to 92 passages. In the presence of activin A, exendin-4, and retinoic acid, these cells expressed pancreatic cell markers, including insulin, Pdx1, pan polypeptide, and neurogenin-3 (Baek *et al.*^2012^).

Tissue damage caused in the salivary gland by ligation of its main ducts leads to the disappearance of acinar cells and intensified proliferation of ductal cells. A population of cells isolated from the ligated rat salivary glands and placed in culture proved to acquire an epithelium-like morphology. When transplanted into the liver via the portal vein, the cells from such a culture integrated with recipient hepatocytes and began to produce albumin. In culture grown in collagen type 1-coated dishes, these cells formed clusters of two major types: one of cells positive for alpha-fetoprotein and/or albumin (the hepatic cluster), and the other of cells positive for glucagon and/or insulin (the pancreatic cluster). In laminin-coated dishes, these cells selectively differentiated into hepatic-type cells. Thus, these cells had characteristics of tissue stem/progenitor cells and were able to differentiate into cells of endodermal lineages (Okumura *et al.*^2003^). Similar results were obtained when Sca-1^+^/c-Kit^+^ mouse submandibular SGC were cultured on matrigel (Hisatomi *et al.*^2004^). These cells acquired the ability to produce albumin and, when transplanted into the portal vein of mice, integrated into hepatic cords and expressed albumin and alpha-1-antitrypsin.

Progenitor cells from the interstitium of human salivary glands were found to be capable to transdifferentiate into cells with a pancreatic endocrine phenotype. In spheroid culture, these cells produced insulin and C-peptide and expressed early islet differentiation factor Nkx6.1, insulin, pro-endocrine factor neurogenin-3, and ductal cell marker cytokeratin 19. Spheroids were also able to release insulin in response to glucose (Sato *et al.*^2007^).

Despite numerous publications on the differentiation potential of SGC, the molecular features of these cells have not been studied sufficiently. Therefore, we decided to perform a side-by-side comparative analysis of cultured submandibular SGC and liver progenitor cells (LPC) from postnatal mice. Such an analysis can provide a deeper insight into what SGC have in common with cells of endodermal origin and help in developing techniques to direct cell differentiation into different endodermal lineages.

## Results

### Cell cultures

**Figure 1:**
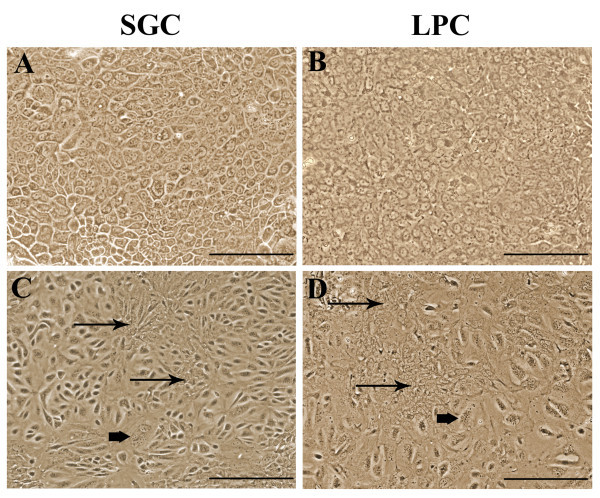
**The morphology of mouse salivary gland and liver progenitor cells (phase contrast microscopy; scale bars = 100 μm): (A) SGC monolayer, primary culture; (B) LPC monolayer, primary culture; (C) SGC monolayer, passage 1; (D) LPC monolayer, passage 1.** Cell heterogeneity is manifested in passage 1 monolayers **(C, D)**, where groups of small actively proliferating cells (thin arrows) and large multinuclear cells (thick arrows) can be seen.

Isolated SGC attached to the collagen-coated plastic dishes within two days to produce colonies of small epithelial-like cells (10–12 μm) with a high nuclear-cytoplasmic ratio, similar to LPC (Figures 1A, B). Primary SGC cultures formed a confluent monolayer on day 5 after isolation, and LPC cultures reached confluence on day 7. Both cell types showed a high proliferative potential; the population doubling time was about 42 h in SGC 63 h in LPC cultures. Under the given culture conditions, the cells sustained 20 passages (until the end of experiments) without losing their phenotype and proliferative potential.

After the first passage, both SGC and LPC in dense cultures acquired the ability to form clusters of small well-proliferating cells and large epithelial-like cells (Figures 1C, D). However, their ability to form differentiated cell clusters disappeared during subsequent passages.

### Salivary gland cells possess immunophenotypic similarities with endodermal cells

**Figure 2:**
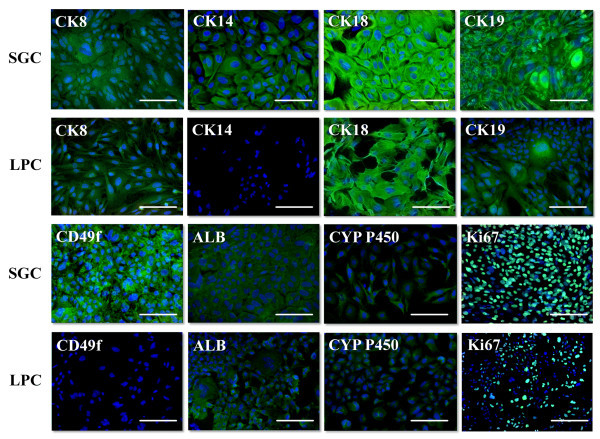
**Immunocytochemical analysis of first-passage salivary gland and liver progenitor cells, fluorescent microscopy.** Cell nuclei were stained with DAPI (blue), the antigens were detected with Alexa Fluor 488-conjugated antibodies (green), scale bars = 100 μm.

**Figure 3:**
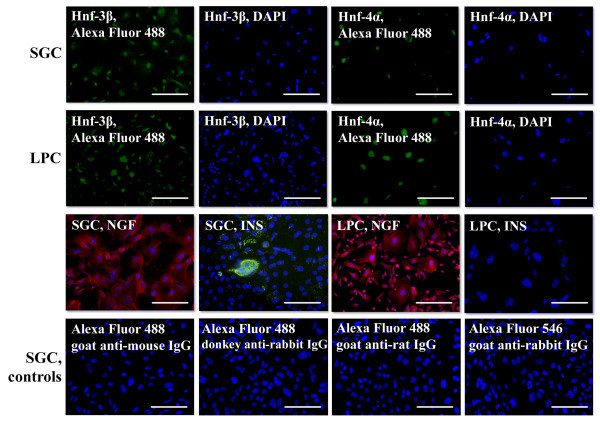
**Immunocytochemical analysis of first-passage salivary gland and liver progenitor cells, fluorescent microscopy.** Cell nuclei were stained with DAPI (blue), the antigens were detected using antibodies conjugated with Alexa Fluor 488 (green) and Alexa Fluor 546 (red), scale bars = 100 μm.

First-passage monolayer cell cultures were immunophenotyped for the markers listed in Table 1. The high proliferative potential of SGC was confirmed by the fact that more than 90% of these cells were positive for the Ki67 marker, with the proportion of Ki67^+^ cells in LPC culture averaging 50% (Figure 2). In both cultures, the cells expressed cytokeratins 8 and 18, typical for glandular cells of endodermal origin, and cytokeratin 19, which is regarded as a marker of epithelial stem/progenitor cells. Intracellular localization of cytokeratins 8 and 18 was similar in both cultures; cytokeratin 19 in LPC was localized near the nucleus, but in SGC is was also detected close to the cell membrane. The expression of ductal cell markers cytokeratin 14 and CD49f was detected in SGC but not in LPC (Figure 2). The cells of both cultures showed slight positive staining for albumin and cytochrome P450 1A1 (Figure 2) and expressed nerve growth factor (NGF) (Figure 3). Moreover, differentiated SGC cultures expressed insulin, while LPC were insulin-negative (Figure 3). Both types of cultured cells were positive for endoderm-enriched hepatocyte nuclear factors Hnf-3β and Hnf-4α (Figure 3).

**Table 1:** **Antibodies used in the study (asterisks indicate antibodies used for flow cytometry)**

Antibody	Manufacturer, catalog number	Species	Dilution
**Primary antibodies**			
AFP*	R&D, # MAB1368	mouse anti-human/mouse IgG1	1:200
ALB*	R&D, # MAB1455	mouse anti-human/mouse IgG2a	1:200
CD29*	Millipore, # FCMAB269F	rat anti-mouse IgG2aκ	2 μl per 10^6^ cells
CD49f*	Millipore, # MAB1378	rat anti-human/mouse IgG2a	1:10
CD45*	Millipore, # FCMAB126F	mouse anti-human/mouse IgG2bκ	10 μl per 10^6^ cells
CD90*	Millipore, # CBL1500F	mouse anti-rat/mouse IgG1	10 μl per 10^6^ cells
CD133*	Millipore, # MAB4310X	rat anti- mouse IgG1κ	1:200
CK8	AbCam, # ab59400	rabbit anti-human/rat/mouse IgG	1:500
CK14	Chemicon, # CBL197	mouse anti-human IgG3	1:100
CK18	Millipore, # MAB3234	mouse anti-human/mouse IgG1	1:100
CK19*	AbCam, # ab15463-1	rabbit anti-human/ mouse IgG	1:100
CYP P450 1A1	Millipore, # AB1258	rabbit anti-human	1:100
c-Kit*	Millipore, # CBL1360	rat anti- mouse IgG2bκ	1 μl per 10^6^ cells
c-Met*	Millipore, # MAB3729	mouse anti-human/mouse IgG1	1:500
EpCAM*	Abcam, # ab32392	rabbit anti-human/rat/mouse IgG	1:500
Hnf-3β	Millipore, # 07-633	rabbit anti-human/rat/mouse IgG	1:200
Hnf-4α	Santa Cruz, # SC-8987	rabbit anti-human/rat/mouse IgG	1:200
INS	R&D, # MAB1417	rat anti-human/bovine/mouse IgG2A	1:200
Ki67	Millipore, # MAB4190	mouse anti-human IgG1	1:200
NGF	Millipore, # 04-1111	rabbit anti-human/rat/mouse IgG	1:200
Sca-1*	Millipore, # FCMAB224F	rat anti-mouse IgG2aκ	2 μl per 10^6^ cells
**Secondary antibodies**			
Alexa Fluor® 488*	Invitrogen, # A-21206	donkey anti-rabbit IgG (H + L)	1:1000
Alexa Fluor® 546	Invitrogen, # A-11035	goat anti-rabbit IgG (H + L)	1:1000
Alexa Fluor® 488*	Invitrogen, # A-11029	goat anti-mouse IgG (H + L)	1:1000
Alexa Fluor® 488*	Invitrogen, # A-11006	goat anti-rat IgG (H + L)	1:1000

### Salivary gland cells express mRNA of some liver-enriched genes

**Figure 4:**
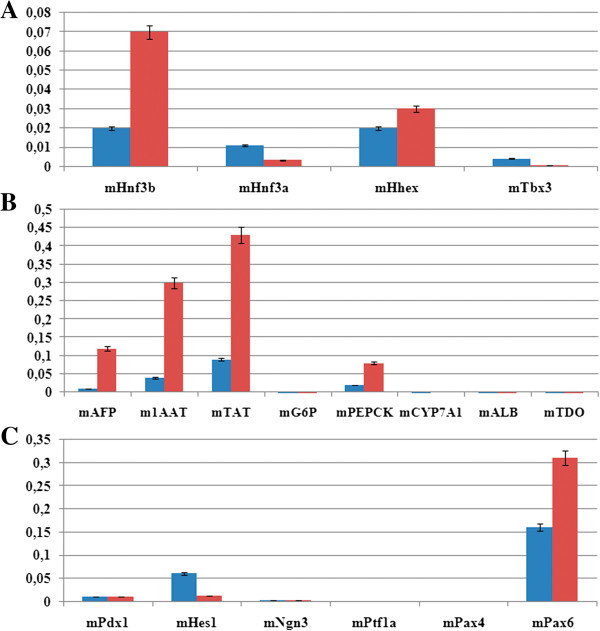
**Quantitative RT-PCR analysis of first-passage salivary gland and liver progenitor cells for mRNAs of different cell markers (relative to GAPDH mRNA expression level taken as 1).** Blue and red bars refer to SGC and LPC, respectively. **(A)** Liver-enriched transcription factors; **(B)** liver-specific cell differentiation markers; **(C)** pancreas-enriched transcription factors.

Comparative qRT-PCR analysis demonstrated that the mRNA expression of transcription factors involved in hepatic differentiation is active in both cell cultures. The expression of the early endoderm marker Hnf-3β was increased 3.5-fold in LPC cultures, while the liver- and pancreas-enriched transcription factor Hnf-3α was expressed in SGC at higher level than in hepatic cells. These factors are homologous and recognize the same DNA sequences, but each of them also has specific functions. Hnf-3α plays a pivotal role in pancreatic cell function, while Hnf-3β is essential for early liver and pancreas development (Su *et al.*^2009^). The mRNA expression of Hhex, a factor involved in processes of cell migration and morphogenesis, was at a comparable level in both cultures. The expression of tumor suppressor 19^ARF^ Tbx3 was ten times higher in SGC than in LPC, remaining at a generally low level (Figure 4A).

The mRNAs for proteins characteristic of hepatocytes were expressed in both cultures at a relatively low level (Figure 4B). These were mainly the markers of early stages of liver cell differentiation (alpha-fetoprotein, alpha-1-antitrypsin). The level of their expression was approximately ten times higher, and that of tyrosine aminotransferase mRNA was five times higher in LPC than in SGC cultures. The mRNAs for proteins typical of later differentiation stages (albumin, G6P, cytochromes, TDO) were expressed only slightly, indicating that the studied endodermal cells are in an undifferentiated state.

The expression of mRNAs for pancreas-enriched transcription factors was also analyzed (Figure 4C). In both cultures, the spontaneous expression of Pdx1 mRNA was detected at a low level. The expression of Ngn3 mRNA was also low, possibly due to the presence of Hes1, an Ngn3 antagonist. A relatively high expression level in SGC and LPC cultures was detected for Pax6, which is also involved in the pancreatic alpha and beta cell differentiation. The level of mRNA for mPtf1a, which initiates acinar cells differentiation, was close to zero.

### Salivary gland and liver cells are similar in surface cell markers expression but differ in transcription factors gene expression

Deep-sequencing transcriptome analysis was performed in the first-passage monolayer cell cultures. As a result, more than 28 000 transcripts were identified. To gain a deeper understanding of similarity between SGC and LPC, we compared the corresponding mRNA expression profiles of genes encoding (1) liver-specific surface antigens and cell markers characteristic of different stages of liver development (Table 2), (2) various transcription factors (Table 3), and (3) cytokines involved in liver cell differentiation.

**Table 2:** **Expression analysis of genes for surface antigens and cell differentiation markers by deep sequencing in first-passage SGC and LPC cultures (data are normalized for the total number of reads)**

Gene	Read number per mRNA (x10^−6^)		SGC/LPC ratio of gene read numbers
	SGC	LPC	
**EpCAM**	499	2.336	214
**NCAM**	4.613	9.466	0.5
**CD133**	0.686	0.121	5.7
**CD44**	174	90.168	2
**Claudin 3**	79.954	5.218	15
**Claudin 7**	136	0.485	280
**Shh**	0.405	0.212	2
**CK19**	54.861	33.525	1.6
**Dlk-1**	0.218	0	-
**c-Kit**	0.686	1.123	0.6
**Sca-1**	742	22.087	34
**CD90**	30.579	38.318	0.8
**LGR5**	1.403	0.728	1.9
**AFP**	1.091	2.245	0.5
**ALB**	0.03	0.212	0.14
**c-Met**	99.436	19.296	5
**CYP P450 3A13**	2.213	0.698	3
**PEPCK**	0.187	0.212	1
**TDO**	0.094	0.061	1.5
**ALB**	0.03	0.212	0.14
**1-AAT**	0.125	0.121	1
**CK7**	1079.738	14.532	74
**CK9**	0.218	0	-
**CYP P450 7A1**	0.094	0.061	1.5

**Table 3:** **Expression analysis of genes for transcription factors by deep sequencing in first-passage SGC and LPC cultures (data are normalized for the total number of reads)**

Gene	Read number per mRNA (x10^−6^)		SGC/LPC ratio of gene read numbers
	SGC	LPC	
**Gata4**	0.125	15.989	0.008
**Gata6**	0.125	17.597	0.007
**Hnf-1α**	0.062	1.244	0.05
**Hnf-1β**	0.031	9.132	0.003
**Hnf-3α**	41.832	2.457	17
**Hnf-3β**	0.405	4.581	0.1
**Hnf-3γ**	0	0.061	0
**Hnf-4α**	0.03	13.106	0.0002
**Hnf-4β**	0	0	-
**Hnf-4γ**	0.187	0.303	0.6
**Sox7**	0.53	5.097	0.1
**Sox9**	172.626	11.165	15.5
**Sox17**	0	1.001	0
**Hhex**	9.351	31.886	0.3
**Tbx3**	5.891	1.517	4
**Prox1**	0.468	1.365	0.3
**OC-2**	0.249	16.565	0.02
**Notch**	82.23	67.656	1.2
**Jag1**	135.283	93.566	1.4

In both cell cultures, the expression of epithelial cell adhesion marker EpCAM was detected. Its level was 200 times higher in SGC than in LPC cultures, which could be evidence for a higher proliferative potential of SGC and their active involvement in the processes of cell layer formation. Moreover, SGC and LPC cultures expressed mRNAs of hepatic stem cell markers NCAM, c-Kit, CD44, and CK19. The expression of mRNAs for CD113 and claudins 3 and 7, which mark the oval cells, was higher in SGC. These cells, unlike LPC, also showed slight expression of Dlk-1. All these data are indicative of considerable phenotypic similarity between SGC and LPC in the expression of cell surface markers. Both cell cultures fairly strongly expressed the WNT-target gene LGR5, which marks progenitor cells involved in organ regeneration. It has been shown that damage-induced liver LGR5^+^ cells can generate functional hepatocytes and bile ducts when transplanted *in vivo,* thereby contributing to liver regeneration (Huch *et al.*^2013^).

In both cultures, differentiated cell markers 1AAT, ALB, TDO, and PEPCK were expressed at a low level. Hepatocyte-specific cytochrome P450 3A13 was more strongly expressed in SGC, but its expression could be stimulated by cell adaptation to the *in vitro* culture conditions.

Cholangiocyte marker CK9 was expressed at a low level only in SGC. Ductal cytokeratin 7 was detected in both cell lines, but its expression in SGC was much higher. The expression of cholangiocyte-specific cytochrome P450 7A1 was very low in both cultures. All these data suggest that SGC and LPC cultures were heterogeneous and comprised cells at different stages of differentiation, with the proportion of more differentiated cells being relatively low.

The expression of transcription factors Gata4 and Gata6, which are involved in early endoderm development, was approximately 100 times lower in SGC than in LPC cultures (Table 3). These factors are necessary for activation of liver-specific hepatocyte nuclear factors and initiation of hepatic differentiation. Factors Hnf-1β, Hnf-3β and Sox7 in SGC were also weakly expressed, and Sox17 was not detected at all, while the expression levels of early endoderm markers Sox9 and Hnf-3α were 17 times and 15 times higher, respectively, than in LPC. The expression of genes *Hhex* and *OC-2,* which are responsible for cell migration and liver morphogenesis, was more active in LPC. However, the expression of Tbx3, which plays an important role during hepatoblasts differentiation, was four times higher in SGC than in LPC.

Liver-enriched transcription factors, including hepatocyte nuclear factors (Hnf-1, Hnf-3, Hnf-4, Hnf-6), play a key role in the maintenance of hepatocyte-specific transcription. The expression of Hnf-1 factors required for the differentiation and functioning of hepatocytes (Hnf-1α) and cholangiocytes (Hnf-1β) was 20 and 295 times lower, respectively in SGC than in LPC. The expression of Hnf-3α in SGC was 17 times higher, while that of Hnf-3β was about 10 times lower than in LPC. The Hnf-3γ expression was not detected in SGC cultures, and the expression of Hnf-4α, which is the key transcription factor in the liver, was 400 times lower than in the LPC.

These results suggest that SGC express some transcription factors characteristic of early endoderm (Sox9 and Hnf-3α) but do not express at a high level the entire complex of genes involved in liver cell differentiation.

The expression of mRNAs for retinoic acid receptor (RArβ), retinoic acid binding protein I (Crabp1) and the gene 6 (Stra6) stimulated by retinoic acid was higher in SGC cultures. It is known that retinoic acid plays an important role in pancreas development and is also important for the induction of Pdx1 expression. Thus the high expression of retinoic acid signaling genes and Hnf-3α makes SGC similar to the exocrine cells of the pancreas.

In contrast to LPC, SGC showed a high expression level of the desmosome and tight junction proteins (Jup, Dsc2, Tjp1, Dsp) and of proteins involved in the vesicular transport system and the establishment of cellular asymmetry (Mboat1, Vps29, Snap23, Cplx2). These differences appear to be accounted for by the barrier and secretory functions carried out by the cells of salivary gland ducts. The expression of proteins responsible for the contractile properties of cells (Tpm1, Cnn3, Myo1b) was also higher in SGC than in LPC (data not shown).

The mRNAs for enzymes involved in metabolism of carbohydrates (Gsk3a, G6pc3) and urea (Arg1) were expressed at a low level in both cell cultures, and the mRNAs for proteins involved in cholesterol metabolism (Pltp, Ch25h) were detected only in LPC.

Differences between SGC and LPC were also observed in the expression of mRNAs for extracellular matrix components, collagens and laminins. Thus, specific mRNAs for laminins α3, α5, β3, γ2, and B1 were detected in SGC, while LPC contained mRNAs for laminins γ1 and B1; SGC generally expressed mRNAs for collagens typical of the basal membrane (8α1, 4α1, 4α2), while collagens expressed by LPC (1α1, 1α2, 3α1, 4α1, 4α2, 5α1) are common for the extracellular matrix of the liver. In addition, both cell cultures expressed fibronectin. As for the mRNAs of integrins, those encoding the integrin subunits of laminin receptors (α3, α6, αv, β1, β2, β4) were generally detected in SGC, while mRNAs for the integrin subunits of fibronectin receptors (α5, α6, αM, αv, β1, β2, β4-low, β5) were found mainly in LPC. Thus, judging from the expression pattern of integrin mRNAs, it appears that receptors to laminin composed of integrins α6β1 and α3β1 are the major receptor type on SGC; in addition, these cells may contain laminin receptor α6β4 and a receptor for vitronectin, fibronectin, fibrinogen and laminin αvβ1. The main receptors on LPC are probably composed of integrins αMβ2 (a fibrinogen receptor), α5β1 (a fibronectin receptor), and αvβ1 (a receptor to vitronectin, fibronectin, fibrinogen and laminin).

Both SGC and LPC cultures showed a high-level expression of mRNAs from the Notch gene family and Jag1 gene, which account for switching from hepatocyte to cholangiocyte differentiation and are important for ductal structure formation and cell maintenance in an undifferentiated state. The mRNAs of HGF and its receptor c-Met were also detected in both cell types. It is known that HGF stimulates branching morphogenesis in salivary gland ducts and proliferation of hepatic cells and maintenance of hepatocyte differentiation in the liver.

Oncostatin M and its receptors were expressed in both cell cultures at a fairly high and roughly comparable level. The mRNA of interleukin 6, which initiates hepatic cells proliferation, was stronger in LPC cultures. Neither SGC nor LPC expressed FGF4, which plays a key role at the initial stages of hepatic differentiation, although a high expression of mRNA for the FGF4 receptor was detected in LPC (but not in SGC). In addition, FGF1 mRNA was expressed in SGC, and bFGF and FGF7 (KGF) mRNAs were detected in LPC cultures.

In general, the mRNA expression patterns in SGC and LPC cultures indicate that they possess characteristics of progenitor cells. In particular, both cell types express the set of transcription factors, receptors, and cytokines that help to maintain them in an undifferentiated state.

### Salivary gland cell culture consists mostly of EpCAM^+^ ductal progenitor cells

On the whole, flow cytometry data confirm the results of immunocytochemical and genome-wide transcriptome analyses. Both cell cultures contained high percentages of ALB^+^, AFP^+^, CK19^+^, and c-Met^+^ cells (Table 4, Additional file 1: Figure S1). More than 90% of cells in SGC cultures expressed integrin subunits of laminin receptor (CD29 and CD49f), while the fractions of CD29^+^ and CD49f^+^ cells in LPC cultures were 52% and about 2%, respectively. SGC cultures contained no CD45^+^ cells, but we detected approximately 3% of such cells in LPC cultures.

**Table 4:** **Flow cytometry analysis of first-passage SGC and LPC cultures**

Cell marker	SGC	LPC
**AFP**	73.3 ± 4.9	71.9 ± 2.5
**ALB**	82.6 ± 2.7	89.8 ± 4.8
**c-Met**	67.4 ± 6.3	69.9 ± 0.7
**CK19**	93.8 ± 2.1	70.2 ± 4.8
**CD29**	93.3 ± 1.0	52.2 ± 3.2
**CD49f**	87.8 ± 3.4	1.7 ± 0.2
**CD45**	0.1 ± 0.1	3.0 ± 0.8
**CD90**	3.7 ± 0.7	21.1 ± 2.3
**CD133**	10.0 ± 1.6	31.1 ± 0.7
**c-Kit**	2.1 ± 0.2	1.9 ± 0.5
**Sca-1**	2.2 ± 0.1	10.9 ± 1.3
**EpCAM**	69.1 ± 2.4	12.3 ± 0.4

Background fluorescence level was 0.2 ± 0.1%.

**Figure 5:**
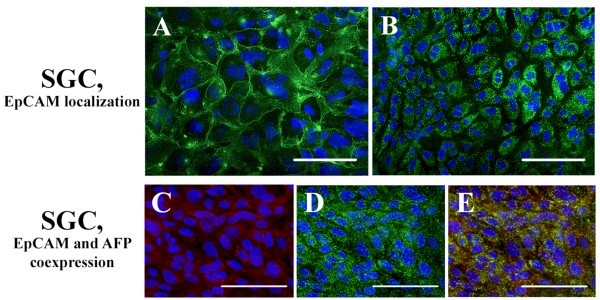
**Immunocytochemical analysis of EpCAM expression features in first-passage SGC culture.** Cell nuclei were stained with DAPI (blue), EpCAM was stained with Alexa Fluor 488-conjugated antibodies (green), and alpha-fetoprotein was stained with Alexa Fluor 546-conjugated antibodies (red); scale bars = 100 μm: **(A)** membrane localization of EpCAM (paraformaldehyde fixation without Triton X-100); **(B)** cytoplasmic localization of EpCAM (fixation with 70% ethanol); **(C)** alpha-fetoprotein; **(D)** EpCAM; **(E)** coexpression of alpha-fetoprotein and EpCAM.

The cells expressing stem cell markers CD90, CD133, c-Kit, and Sca-1 were found in both SGC and LPC cultures, but their fraction in LPC cultures was usually higher. EpCAM marker, detected in SGC cultures by transcriptome analysis, was found in 70% of cells, while in LPC cultures this marker was detected in only 12% of cells. Some specific features of EpCAM localization were observed in SGC culture, where this marker proved to be coexpressed with alpha-fetoprotein (Figures 5C–E). Coexpression of EpCAM and alpha-fetoprotein was previously observed in embryonic hepatoblasts (Schmelzer *et al.*^2007^), but in the adult liver and SGC cultures, unlike in embryonic hepatoblasts, we observed cytoplasmic as well as membrane localization of EpCAM (Figure 5A, B).

Thus, both SGC and LPC cultures comprise cells expressing stem cell markers. The SGC culture appears to be a relatively homogeneous progenitor-enriched population formed by cells of ductal origin: about 90% of SGC are CD29^+^, CD49f^+^, and CK19^+^ cells, and about 70% express EpCAM. The LPC culture is more heterogeneous and consists mainly of EpCAM^+^ as well as CK19^+^ and AFP^+^ progenitor cells.

## Discussion

In general, cultured SGC are small epithelial-like cells (10–12 μm) with a high nuclear-cytoplasmic ratio that are capable of self-renewal over at least 20 passages, with the population doubling time being about 40 h. Approximately 90% of cultured SGC express ductal markers CD49f, CD29 and CK19. These cells are also positive for epithelial markers CK 8, 18, 19 and endoderm-enriched markers Hnf-3α, Hnf-3β, and Sox9. Similar to liver progenitor cells, cultured SGC express specific progenitor cell markers EpCAM, NCAM, c-Kit, CD44, and CD133, with high-level EpCAM expression being observed in 70% of these cells. A major proportion of SGC (70–80%) are also positive for albumin, alpha-fetoprotein, and c-Met. Thus, cultured SGC express markers of progenitor cells originating from salivary gland ducts.

The culture of liver cells is more heterogeneous. Only about 3% of them express hematopoietic marker CD45, and 12% express EpCAM. On the other hand, a significant proportion of these cells express albumin, alpha-fetoprotein, CK19, and c-Met.

In the postnatal liver, EpCAM marks stem cells of the canals of Hering and progenitors of bile ducts (Schmelzer *et al.*^2007^; Trzpis *et al.*^2007^). EpCAM^+^ liver cells are positive for albumin (weakly) and cytokeratin 19 but negative for alpha-fetoprotein. These cells are regarded as candidates for postnatal hepatic stem cells with the ability to differentiate into bipotent (oval) alpha-fetoprotein-positive cells (Schmelzer *et al.*^2007^). Unlike the liver cells, EpCAM^+^ cells of salivary glands possess a unique gene expression profile, being also positive for alpha-fetoprotein. In the body, EpCAM is expressed in the simple epithelium and acts as an antagonist of E-cadherin. EpCAM regulates the processes of cell layer organization, cell migration, and proliferation (Trzpis *et al.*^2007^). In the salivary glands, a high level of EpCAM expression can be associated with active self-renewal processes.

The CD49f marker is common to ductal cells of rat, mouse, and human salivary glands (Sato *et al.*^2007^). The primary culture of CD49f^+^ cells from the human submandibular salivary gland was found to contain about 2% of Thy-1^+^ cells. Single purified salivary gland progenitor cells in culture expressed intracellular laminin, CD49f, Thy-1, and NGF receptor p75 (p75^NGFR^). These cells could be passaged at least 15 times without losing their ability to differentiate into insulin- and albumin-producing cells. *In vivo,* Thy-1^+^/CD49f^+^ cells in the submandibular salivary gland were found in the stroma, particularly in the periductal area (Sato *et al.*^2007^).

Hisatomi et al. (2004) sorted the Sca-1^+^/c-Kit^+^ progenitor cell fraction out of adult mouse submandibular salivary glands. Cultured Sca-1^+^/c-Kit^+^ cells expressed CD49f and alpha-fetoprotein. In spheroid cultures, these progenitors differentiated into a pancreatic endocrine lineage in the presence of glucagon-like peptide-1; when cultured in matrigel, they differentiated into a hepatic lineage (Hisatomi *et al.*^2004^). Progenitor cells isolated from swine submandibular salivary glands were CD49f-positive. After forming three-dimensional structures, these cells expressed insulin and albumin. Differentiated in spheroid culture cells were able to release insulin (Matsumoto *et al.*^2007^).

On the whole, SGC and LPC cultures have shown a similar expression pattern of surface progenitor cell markers but proved to differ in the expression of transcription factors. In SGC, early endodermal markers Hnf-3α and Sox9 are expressed at a higher level, while the expression of Gata4 and Gata6, transcription factors necessary for the initiation of hepatic differentiation (Murry and Keller 2008; Soto-Gutierrez *et al.*^2008^; Zaret and Grompe 2008), is 100 times lower than in LPC. Accordingly, the expression level of regulatory factors required for the differentiation of hepatocytes (Hnf-1α, Hnf-3β, and Hnf-4α) is lower in SGC than in LPC. Hnf-3α and Hnf-3β play an important role in the development of the liver and pancreas. The expression of Hnf-3β is higher in the postnatal liver, while that of Hnf-3α is higher in the postnatal pancreas (Su *et al.*^2009^). Thus, the expression patterns of the hepatocyte nuclear factors in SGC cultures and in the cells of the pancreas are similar. Moreover, the similarity between SGC cultures and pancreatic cells also concerns the active expression of intracellular components of the retinoic acid signaling, which is of importance in the induction of Pdx1 expression (Zaret and Grompe 2008; Katsumoto and Kume 2011).

The high expression level of cytokeratins 8, 18, and 19, alpha-fetoprotein, c-Met, and endoderm-enriched transcription factors Hnf-3α and Sox9 makes cultured submandibular SGC cells phenotypically similar to endodermal cells. It is noteworthy in this context that the intercalated and striated ducts of adult human submandibular salivary glands were reported to be alpha-fetoprotein-positive (Tsuji and Nagai 1993). Moreover, preproinsulin I and II mRNAs were found to be expressed in rat submandibular salivary glands, like in the pancreatic islets (Egea *et al.*^2000^). Taking into account the high ability of SGC to differentiate into endodermal lineages (Okumura *et al.*^2003^; Hisatomi *et al.*^2004^; Matsumoto *et al.*^2007^; Sato *et al.*^2007^; Baek *et al.*^2012^), it was assumed that ductal cells of submandibular salivary glands could have endodermal origin.

Rothova et al. (2012) analyzed the fate of endodermal cells during embryonic oral development in Sox17-2A-iCre/R26R mice. The progeny of Sox17^+^ endodermal cells were not detected in major salivary glands at embryonic day 15.5, providing evidence for the ectodermal origin of these glands in mice (Rothova *et al.*^2012^).

We suggest that similarities of mouse submandibular salivary gland cells and endodermal cells can appear through the community of theirs functions in the alimentary tract. This phenotypic convergence of the salivary gland cells and endodermal cells could be associated with high differentiation potential of salivary gland cells within endoderm. Therefore, the ductal cells of submandibular salivary glands may be a convenient source of cells for cellular therapy of the liver and pancreas.

## Conclusions

Submandibular salivary glands can serve as an alternative cell source for cellular therapy of the liver and pancreas disorders. Cultured submandibular salivary gland cells appear to be a promising for allogeneic or autologous transplantation, because they are easy to isolate and culture. Cultured submandibular salivary gland cells have shown phenotypic convergence with cells of endodermal origin.

## Materials and methods

### Animals

The study was performed with 8- to 15-week-old C57BL/6 male mice kept under standard conditions, with free access to food and water. All animal procedures were carried out in accordance with the Guidelines for Humane Endpoints for Animals Used in Biomedical Research and Regulations for Laboratory Practice in the Russian Federation, under the supervision of the Ethics Committee for Animal Research of the Koltsov Institute of Developmental Biology, Russian Academy of Sciences.

### Cell culture

The mice were sacrificed under chloroform anesthesia, and both submandibular salivary glands and the liver were excised under aseptic conditions and placed in sterile tubes with DMEM/F-12 (1:1) medium (Gibco) containing 40 μg/ml gentamicin. The organs were then dissected to remove blood vessels and connective tissue, minced, and washed twice with phosphate-buffered saline (PBS). The resulting homogenates were incubated in DMEM/F-12 (1:1) with 0.1% type IV collagenase (Sigma) at 37°C for 30–40 min, further homogenized by pipetting, and passed through a filter with 40 μm pore size to separate small cells from larger polyploid cells. The filtered cells were washed in two portions of the culture medium using “gentle” centrifugation (2 min at 100 *g*), plated in collagen type 1-coated culture dishes (Corning) at a density of 5 × 10^3^ cells/cm^2^, and cultured in DMEM/F-12 (1:1) with 10% fetal bovine serum (FBS) (HyClone), 2 mM glutamine (Gibco), 1% insulin-transferrin-selenium supplement (ITS; Invitrogen), and 10 ng/ml epidermal growth factor (EGF). The medium was changed every day during the first 5 days and every 3 days in the subsequent period. For passaging cells were harvested using 0.25% trypsin, and plated in a 1:3 dilution in new dishes.

### Immunocytochemical analysis

Cell cultures grown for 3–4 days were fixed with 4% paraformaldehyde for 10 min, permeabilized with 0.1% Triton X-100 and incubated in 1% bovine serum albumin solution in PBS at room temperature for 30 min. Primary and secondary antibodies (Table 1) were diluted in PBS as recommended by the manufacturer. The cultures were incubated with primary antibodies for 1 h at 37°C (or overnight at 4°C), washed with three portions of PBS, and incubated with secondary antibodies for 40 min at 37°C. Thereafter, the cultures were washed with three portions of PBS, 10 min each, and counterstained with DAPI (added to the last portion of PBS).

### RNA extraction

Total RNA was extracted from the cells using an AllPrep DNA/RNA Mini Kit (Qiagen) as recommended by the manufacturer and quantified using a Quibit minifluorometer and an RNA Assay Kit (Invitrogen). A 500-ng aliquot of total RNA was taken to synthesize cDNA using a Superscript II kit (Invitrogen) and random primers according to the manufacturer’s protocol. Five-hundred nanograms of total RNA were used in reaction.

### Quantitative RT-PCR

Quantitative real-time PCR (qRT-PCR) was performed using EVA Green kit (Syntol) and CFX96 system (BioRad). The amplification procedure included DNA polymerase activation at 95°C for 5 min followed by 40 cycles of denaturation at 95°C (30 s), annealing at 57–59°C (30 s), and elongation at 72°C (45 s). The annealing temperature was varied with regard to the primer’s melting point (Table 5). Fluorescence detection in FAM channel and primary processing of the results were performed automatically by the system’s software. Samples were run in triplicate and normalized with reference to GAPDH.

**Table 5:** **Primers used in qRT-PCR**

Primer	Gene	Nucleotide sequence	Amplicon size	Melting point
			bp	°C
**Control**				
mGAPDH	Glyceraldehyde-3-phosphate dehydrogenase	5′- AGG TCG GTG TGA ACG GAT TTG -3′	95	62.6
		5′- GGG GTC GTT GAT GGC AAC A -3′		62.6
**Hepatic markers**				
m1AAT	Alpha-1-antitrypsin	5′- CTC GTC CGC TCA CTA AAC AAG -3′	248	60.7
		5′- GCT GTC TGA GAG TCA AGG TCT T -3′		61.3
mAFP	Alpha-fetoprotein	5′- CCA TCA CCT TTA CCC AGT TTG T -3′	101	60.2
		5′- CCC ATC GCC AGA GTT TTT CTT -3′		60.6
mALB	Albumin	5′- TGC TTT TTC CAG GGG TGT GTT -3′	167	62.4
		5′- TTA CTT CCT GCA CTA ATT TGG CA -3′		60.2
mCYP7A1	Cytochrome P450, family 7, subfamily a, polypeptide 1	5′- AAC GGG TTG ATT CCA TAC CTG G -3′	126	62.0
		5′- GTG GAC ATA TTT CCC CAT CAG TT -3′		60.0
mG6P	Glucose-6-phosphatase	5′- CGA CTC GCT ATC TCC AAG TGA -3′	208	61.0
		5′- GGG CGT TGT CCA AAC AGA AT -3′		60.9
mPEPCK	Phosphoenolpyruvate carboxykinase 1	5′- TGA CAG ACT CGC CCT ATG TG -3′	153	61.0
		5′- CCC AGT TGT TGA CCA AAG GC -3′		61.4
mTAT	Tyrosine aminotransferase	5′- AGC CGA ATC CGA ACA AAA CC -3′	146	60.9
		5′- GCC GAT AGA TGG GGC ATA GC -3′		61.3
mTDO	Tryptophan 2,3-dioxygenase	5′- AAT CCA TGA CGA GCA CCT ATT CA -3′	140	61.4
		5′- TCA CCT TGA GCA TGT TCC TCT -3′		60.8
**Liver-enriched transcription factors**				
mHnf3α	Forkhead box A1 (Foxa1)	5′- GGA GTT GAA GTC TCC AGC GTC -3′	157	62.4
		5′- GGG GTG ATT AAA GGA GTA GTG GG -3′		61.7
mHnf3β	Forkhead box A2 (Foxa2)	5′- TCC GAC TGG AGC AGC TAC TAC -3′	176	62.8
		5′- GCG CCC ACA TAG GAT GAC A -3′		
mHhex	Hematopoietically expressed homeobox	5′- CGA GAC TCA GAA ATA CCT CTC CC -3′	162	61.2
		5′- CTG TCC AAC GCA TCC TTT TTG -3′		60.0
mTbx3	T-box 3 (Tbx3), transcript variant 2	5′- TGG AAC CCG AAG AAG ACG TAG -3′	84	61.2
		5′- TAC CCC GCT TGT GAA ACT GG -3′		62.1
**Pancreas-enriched transcription factors**				
mHes1	Hairy and enhancer of split 1	5′- TCA ACA CGA CAC CGG ACA AAC -3′	155	63.0
		5′- ATG CCG GGA GCT ATC TTT CTT -3′		61.0
mNgn3	Neurogenin 3	5′- CCA AGA GCG AGT TGG CAC T -3′	236	62.3
		5′- CGG GCC ATA GAA GCT GTG G -3′		62.5
mPax4	Paired box gene 4	5′- GCA GTG TGA ATC AGC TAG GGG -3′	103	62.5
		5′- CAG GGT CGC ATC CCT CTT ATT -3′		61.3
mPax6	Paired box gene 6	5′- AAC AGT CAC AGC GGA GTG AAT -3′	196	61.7
		5′- ACA CAA CCG TTG GAT ACG TTT T -3′		60.7
mPdx1	Pancreatic and duodenal homeobox 1	5′- CCC CAG TTT ACA AGC TCG CT -3′	117	62.2
		5′- CTC GGT TCC ATT CGG GAA AGG -3′		62.7
mPtf1a	Pancreas specific transcription factor, 1a	5′- GCT ACA CGA ATA CTG CTA CCG -3′	134	60.3
		5′- CGC AGC AAT AGC TGA CGT TG -3′		62.0

### Gene expression analysis by the deep sequencing

Isolation of mRNA, cDNA synthesis, and preparation of libraries for deep sequencing were performed with mRNA Sequencing Sample Preparation Kit (Illumina), NEBNext mRNA Library Preparation Reagent Set for Illumina (New England Biolabs), QIAquick PCR Purification Kit (Qiagen) and MinElute Gel Extraction Kit (Qiagen) according to the standard protocols. The sequencing procedure (single-end 72 nucleotide reads with Sequencing Control Software (SCS) v. 2.10 and Real Time Analysis (RTA) software v. 1.8) was carried out in a Genome Analyzer IIx (Illumina) as recommended by the manufacturer. The FASTQ Illumina reads have been filtered with fastq_quality_filter to select the reads with a quality of 25 or higher for each letter. These reads were aligned to the whole mouse transcriptome sequences (ftp://ftp.ncbi.nih.gov/genomes/M_musculus/RNA/) with the BWA program using parameters “bwa index -a is” and “bwa aln -N -t 8”. The results were used to calculate the transcripts reads coverage statistics for each RNA sample. The relative abundance of each mRNA was calculated in Microsoft Excel as the number of reads aligned to this mRNA divided by the total number of reads aligned to the mouse transcriptome.

### Flow cytometry

First-passage cells were harvested using trypsin and thoroughly pipetted to prepare single-cell suspensions in PBS with 2% FBS. Samples of the suspensions (1 × 10^6^ cells) were then incubated with primary antibodies (Table 1) for 60 min at room temperature in the dark and washed in three portions of PBS, 10 min each. If primary antibodies were fluorochrome-conjugated, the cells were fixed with 1% paraformaldehyde for 5 min in the dark, washed in three portions of PBS, resuspended in 1 ml of PBS, and analyzed using the Cell Lab Quanta™ SC MPL system (Beckman Coulter). If the primary antibodies were not labeled, the samples after washing were incubated with secondary antibodies for 40 min at room temperature in the dark, and the cells were then fixed and analyzed as described above. Corresponding isotype controls were used, and 10 000 cells per probe were analyzed.

### Statistical analysis

All experiments were performed with three SGC and three LPC cultures obtained from three animals, in three replications per culture. The results were processed statistically using Student’s *t*-test and considered significant at *p* ≤ 0.05.

## Electronic supplementary material

Additional file 1: Figure S1: Flow cytometry analysis of first-passage SGC and LPC. Isotype controls are stained red, target antigens are stained blue. (JPEG 2 MB)

## Abbreviations

DAPI: 4′,6-diamidino-2-phenylindole
EGF: Epidermal Growth Factor
ITS: Insulin-Transferrin-Selenium
LPC: Liver progenitor cells
qRT-PCR: quantitative real-time polymerase chain reaction
SGC: Salivary gland cells.

## References

[CR1] Baek H, Noh YH, Lee JH, Yeon SI, Jeong J, Kwon H (2012). Autonomous isolation, long-term culture and differentiation potential of adult salivary gland-derived stem/progenitor cells. J Tissue Eng Regen Med.

[CR2] Bisgaard HC, Thorgeirsson SS (1991). Evidence for a common cell of origin for primitive epithelial cells isolated from rat liver and pancreas. J Cell Physiol.

[CR3] Cayo MA, Cai J, Delaforest A, Noto FK, Nagaoka M, Clark BS, Collery RF, Si-Tayeb K, Duncan SA (2012). JD induced pluripotent stem cell-derived hepatocytes faithfully recapitulate the pathophysiology of familial hypercholesterolemia. Hepatology.

[CR4] Cheng X, Ying L, Lu L, Galvão AM, Mills JA, Lin HC, Kotton DN, Shen SS, Nostro MC, Choi JK, Weiss MJ, French DL, Gadue P (2012). Self-renewing endodermal progenitor lines generated from human pluripotent stem cells. Cell Stem Cell.

[CR5] Egea JC, Hirtz C, Gross R, Lajoix AD, Traskawka E, Ribes G, de Periere DD (2000). Preproinsulin I and II mRNA expression in adult rat submandibular glands. Eur J Oral Sci.

[CR6] Gvazava IG, Vasiliev AV, Balan OV, Terskikh VV (2011). Study of cell culture of mouse submandibular salivary gland in vitro. Tsitologiia.

[CR7] Hay DC, Fletcher J, Payne C, Terrace JD, Gallagher RC, Snoeys J, Black JR, Wojtacha D, Samuel K, Hannoun Z, Pryde A, Filippi C, Currie IS, Forbes SJ, Ross JA, Newsome PN, Iredale JP (2008). Highly efficient differentiation of hESCs to functional hepatic endoderm requires ActivinA and Wnt3a signaling. Proc Natl Acad Sci U S A.

[CR8] Hisatomi Y, Okumura K, Nakamura K, Matsumoto S, Satoh A, Nagano K, Yamamoto T, Endo F (2004). Flow cytometric isolation of endodermal progenitors from mouse salivary gland differentiate into hepatic and pancreatic lineages. Hepatology.

[CR9] Huch M, Dorrell C, Boj SF, van Es JH, Li VS, van de Wetering M, Sato T, Hamer K, Sasaki N, Finegold MJ, Haft A, Vries RG, Grompe M, Clevers H (2013). In vitro expansion of single Lgr5^+^ liver stem cells induced by Wnt-driven regeneration. Nature.

[CR10] Katsumoto K, Kume S (2011). Endoderm and mesoderm reciprocal signaling mediated by CXCL12 and CXCR4 regulates the migration of angioblasts and establishes the pancreatic fate. Development.

[CR11] Kordes C, Sawitza I, Götze S, Häussinger D (2012). Stellate cells from rat pancreas are stem cells and can contribute to liver regeneration. PLoS ONE.

[CR12] Matsumoto S, Okumura K, Ogata A, Hisatomi Y, Sato A, Hattori K, Matsumoto M, Kaji Y, Takahashi M, Yamamoto T, Nakamura K, Endo F (2007). Isolation of tissue progenitor cells from duct-ligated salivary glands of swine. Cloning Stem Cells.

[CR13] Mizumoto H, Aoki K, Nakazawa K, Ijima H, Funatsu K, Kajiwara T (2008). Hepatic differentiation of embryonic stem cells in HF/organoid culture. Transplant Proc.

[CR14] Murry CE, Keller G (2008). Differentiation of embryonic stem cells to clinically relevant populations: lessons from embryonic development. Cell.

[CR15] Okumura K, Nakamura K, Hisatomi Y, Nagano K, Tanaka Y, Terada K, Sugiyama T, Umeyama K, Matsumoto K, Yamamoto T, Endo F (2003). Salivary gland progenitor cells induced by duct ligation differentiate into hepatic and pancreatic lineages. Hepatology.

[CR16] Rambhatla L, Chiu CP, Kundu P, Peng Y, Carpenter MK (2003). Generation of hepatocyte-like cells from human embryonic stem cells. Cell Transplant.

[CR17] Rothova M, Thompson H, Lickert H, Tucker AS (2012). Lineage tracing of the endoderm during oral development. Dev Dyn.

[CR18] Sato A, Okumura K, Matsumoto S, Hattori K, Hattori S, Shinohara M, Endo F (2007). Isolation, tissue localization, and cellular characterization of progenitors derived from adult human salivary glands. Cloning Stem Cells.

[CR19] Schmelzer E, Zhang L, Bruce A, Wauthier E, Ludlow J, Yao HL, Moss N, Melhem A, McClelland R, Turner W, Kulik M, Sherwood S, Tallheden T, Cheng N, Furth ME, Reid LM (2007). Human hepatic stem cells from fetal and postnatal donors. J Exp Med.

[CR20] Soto-Gutierrez A, Navarro-Alvarez N, Caballero-Corbalan J, Tanaka N, Kobayashi N (2008). Endoderm induction for hepatic and pancreatic differentiation of ES cells. Acta Med Okayama.

[CR21] Su N, Thiaville MM, Awad K, Gjymishka A, Brant JO, Yang TP, Kilberg MS (2009). Protein or amino acid deprivation differentially regulates the hepatic forkhead box protein A (FOXA) genes through an activating transcription factor-4-independent pathway. Hepatologym.

[CR22] Trzpis M, McLaughlin PM, de Leij LM, Harmsen MC (2007). Epithelial cell adhesion molecule: more than a carcinoma marker and adhesion molecule. Am J Pathol.

[CR23] Tsuji T, Nagai N (1993). Production of alpha-fetoprotein by human submandibular gland. Int J Dev Biol.

[CR24] Yi F, Liu GH, Izpisua Belmonte JC (2012). Rejuvenating liver and pancreas through cell transdifferentiation. Cell Res.

[CR25] Zaret KS, Grompe M (2008). Generation and regeneration of cells of the liver and pancreas. Science.

