# Supplementary figures and images for: Comparative analysis reveals similarities between cultured submandibular salivary gland cells and liver progenitor cells
Source: Springerplus. 2014 Apr 9;3:183. doi: 10.1186/2193-1801-3-183 (PMC4000360; doi:10.1186/2193-1801-3-183)

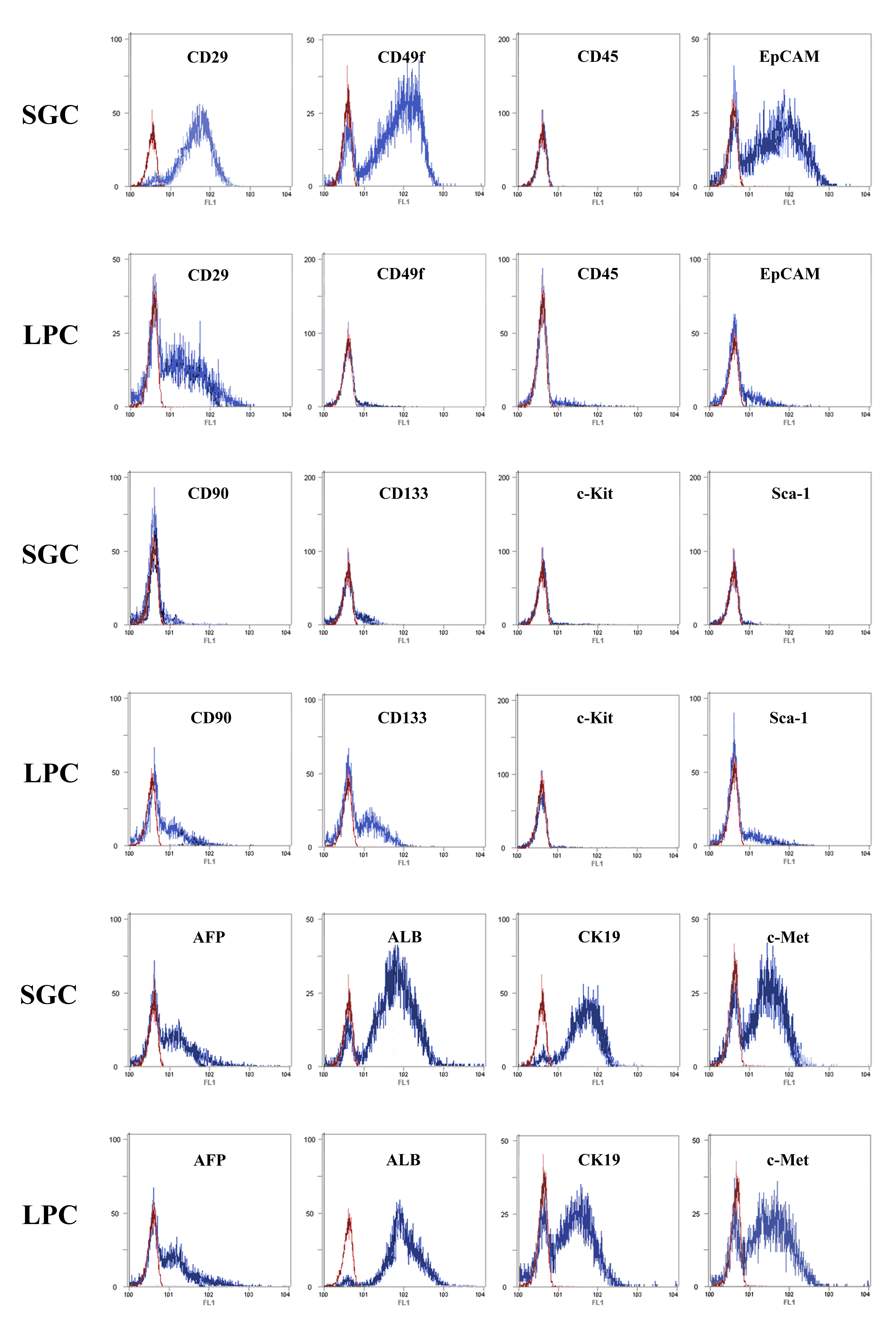

Supplement: Supplementary file 1 — Additional file 1: Figure S1: Flow cytometry analysis of first-passage SGC and LPC. Isotype controls are stained red, target antigens are stained blue. (JPEG 2 MB) [file 40064_2013_890_MOESM1_ESM.jpeg]
